# Molecular switching system using glycosylphosphatidylinositol to select cells highly expressing recombinant proteins

**DOI:** 10.1038/s41598-017-04330-3

**Published:** 2017-06-22

**Authors:** Emmanuel Matabaro, Zeng’an He, Yi-Shi Liu, Hui-Jie Zhang, Xiao-Dong Gao, Morihisa Fujita

**Affiliations:** Key Laboratory of Carbohydrate Chemistry and Biotechnology, Ministry of Education, School of Biotechnology, Jiangnan University, 1800 Lihu Avenue, Wuxi, Jiangsu 214122 China

## Abstract

Although many pharmaceutical proteins are produced in mammalian cells, there remains a challenge to select cell lines that express recombinant proteins with high productivity. Since most biopharmaceutical proteins are secreted by cells into the medium, it is difficult to select cell lines that produce large amounts of the target protein. To address this issue, a new protein expression system using the glycosylphosphatidylinositol (GPI)-anchor was developed. PGAP2 is involved in processing GPI-anchored proteins (GPI-APs) during transport. In *PGAP2* mutant cells, most GPI-APs are secreted into the medium. Here, we established a HEK293 cell line where endogenous *PGAP2* was knocked out and exogenous *PGAP2* was inserted with a *piggyBac* transposon in the genome. Using these cells, human lysosomal acid lipase (LIPA) and α-galactosidase A (GLA) were expressed as GPI-anchored forms (LIPA-GPI and GLA-GPI) and cells expressing high levels of LIPA-GPI or GLA-GPI on the cell surface were enriched. Removal of the *PGAP2* gene by *piggyBac* transposase or FLP recombinase converted LIPA-GPI and GLA-GPI from membrane-bound to the secreted forms. Thus, cells expressing LIPA or GLA in large amounts could be enriched using this approach. The GPI-based molecular switching system is an efficient approach to isolate cells expressing recombinant proteins with high productivity.

## Introduction

The production of recombinant mammalian proteins is of significant interest because of their increasing use in biopharmaceutical purposes and clinical studies^1^. Mammalian culture cells such as Chinese hamster ovary (CHO) cells and human embryonic kidney 293 (HEK293) cells have been used for production of recombinant proteins^2, 3^, because most recombinant mammalian proteins require proper post-translational modifications such as glycosylation for their stability, activity and lower immunogenicity^4, 5^. Most efforts to improve protein expression have focused on vector design, codon optimization, host cell engineering, improved transfection and screening methods, as well as culture medium optimization^6–8^. Despite many efforts to reduce cost, the use of mammalian cell lines for large-scale production of target proteins remains expensive. Furthermore, the selection of cell clones with the highest productivity from the bulk population has been very challenging, time-consuming and very difficult or impossible to achieve^3, 9^. Since most biopharmaceutical proteins are soluble proteins that are secreted into the medium by cells, it is difficult to select a high-producing cell line from the bulk population. To circumvent these problems, a new approach based on simple screening of highly expressing cells is need.

Many proteins present on the cell surface of mammalian cells are attached to the cell surface by a glycosylphosphatidylinositol (GPI) anchor^10, 11^. GPI-anchoring of proteins is conserved among eukaryotes. In mammalian cells, more than 150 GPI-anchored proteins (GPI-APs), including cell-surface receptors, cell adhesion molecules and cell surface hydrolases, have been determined. The GPI anchor is synthesized and transferred to proteins in the endoplasmic reticulum (ER)^11^. Proteins with a GPI-attachment signal are recognized, cleaved and GPI is transferred to the newly exposed C-terminus of proteins by the GPI transamidase complex^10, 12^. GPI-APs are then transported to the plasma membrane through the Golgi apparatus.

**Figure 1:**
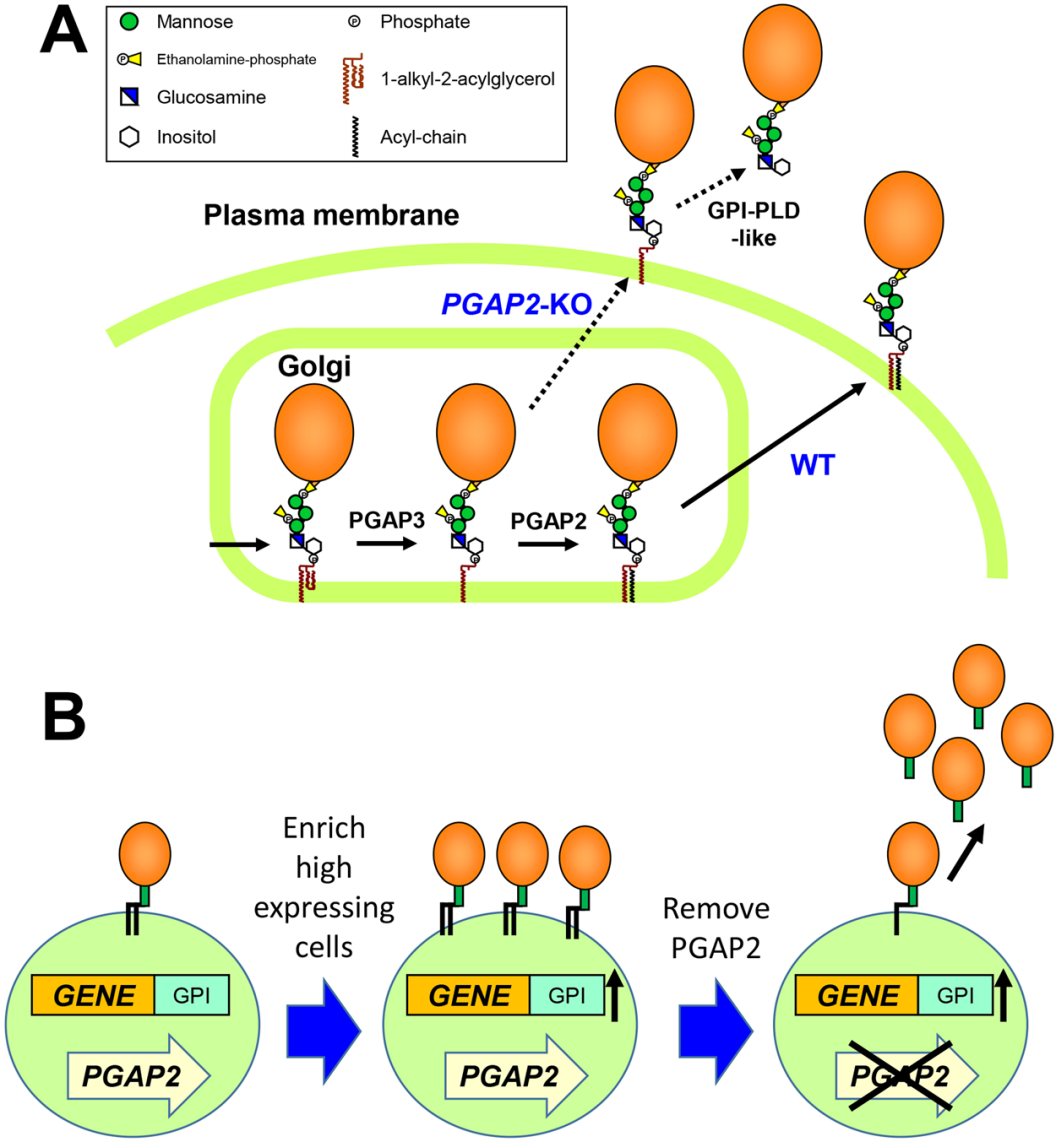
GPI-based protein production system. (**A**) PGAP2 is required for the addition of the saturated acyl chain to the lyso-GPI moiety, which ensures correct expression of GPI-APs on the cell surface. In *PGAP2* mutant cells, lyso-GPI-APs are transported to the cell surface and then most of the GPI-APs are secreted into the medium. Since lyso-forms of GPI-APs are very sensitive to PLD, the GPI structure of secreted proteins become PLD-cleaved forms. (**B**) The GPI-anchoring system ensures the expression of target proteins as GPI-APs on the cell surface. Cells expressing target proteins at a high level can be enriched by selection of the GPI-anchored target proteins. Removal of the *PGAP2* gene results in higher amounts of the target proteins being secreted into the medium.

During the transport, the lipid and glycan of the GPI moiety, which are critical for efficient transport of GPI-APs and association with lipid rafts^13–15^, are remodelled. In mammalian cells, a fatty acid in the *sn*-2 position of the GPI lipid is exchanged in the Golgi apparatus (Fig. 1A), which is named GPI fatty acid remodelling; an unsaturated fatty acid at the *sn*-2 position on GPI is eliminated by PGAP3 (Post-GPI-attachment to proteins factor 3), followed by the transfer of a saturated fatty acid to the *sn*-2 position of the GPI lipid^16, 17^. PGAP2 is required for the latter reaction. In *PGAP2* mutant cells, the fatty acid is not transferred to the *sn*-2 position on the GPI lipid and lyso-forms of GPI-APs are transported to the cell surface^17^. The lyso-forms of GPI-APs cannot stably associate with the plasma membrane and are released from the cell surface to medium. The released GPI-APs are further cleaved by phospholipase D (PLD) like activity^17, 18^.

The GPI anchor is becoming an increasingly important tool for protein expression and cell membrane engineering^19^. When a GPI-attachment signal is added to the C-terminus of secretory proteins, the proteins are expressed as GPI-APs. Therefore, it is possible to express a wide range of recombinant proteins on the cell surface through GPI-anchors^20, 21^. In recent years, several studies have focused on using GPI-anchors for tethering proteins to the cell surface and for their incorporation into extracellular vesicles and virus like particles (VLPs)^20, 22, 23^. Attempts have been made to use GPI-anchored recombinant proteins in the extracellular vesicles and VLPs for biomedical applications, for example, cancer immunology and vaccination^19^.

Here, we developed a mammalian protein expression system using GPI-anchoring. In this system, recombinant proteins are expressed as GPI-anchored forms on the cell surface. Therefore, highly expressing cells can be easily enriched with cell sorters by staining of the GPI-anchored proteins on the cell surface. By removing the *PGAP2* gene, the GPI-anchored recombinant proteins attached to the membrane are released into the medium. Cells highly expressing recombinant lysosomal acid lipase (LIPA) and α-galactosidase A (GLA) were realized using this system. The GPI-based protein expression system was found to be efficient for isolating cells producing recombinant proteins.

## Results

### Generation of PGAP2-KO cells

One of the issues in recombinant protein production in mammalian cells is to select cell lines that are stably expressing recombinant proteins at high levels. Most biopharmaceutical proteins are soluble secretory proteins and are thus secreted by cells into the medium. Although there are cells present in the cell population that are producing high levels of recombinant protein, it is often challenging to select such a high-producing cell line from the bulk cell population. To overcome this issue, we tried to develop a new system to select cells with high protein productivity from a mixture of cells. In this system, we used GPI anchoring of proteins (Fig. 1A). When the secretory target protein is expressed using this system, the proteins are expressed as GPI-anchored forms (Fig. 1B). Therefore, cells expressing high amounts of a target protein can be enriched by sorting the cells for the GPI-anchored form. Finally, the GPI-anchored membrane-bound form can be switched to the secretory form by disruption of the GPI biosynthetic pathway.

**Figure 2:**
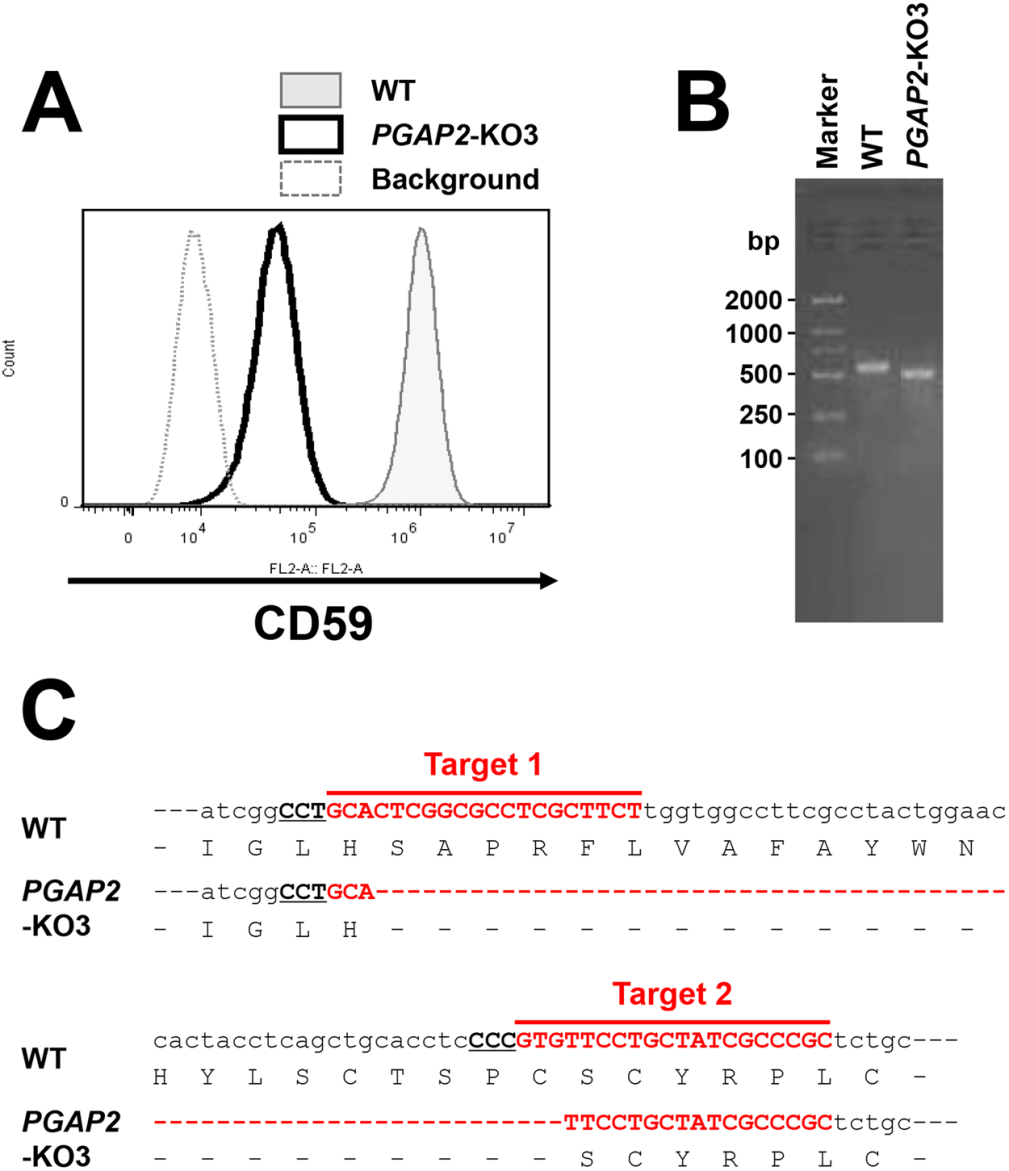
Generation of the *PGAP2*-KO cell line. (**A**) After the *PGAP2* gene was knocked out, the expression of CD59 in a clonal cell line named *PGAP2*-KO3 was analysed. Compared with the wild-type HEK293 (WT), the expression level had decreased significantly in *PGAP2*-KO3 cells. (**B**) Genomic DNA was extracted from wild-type HEK293 (WT) and *PGAP2*-KO3 cells. The knockout region was amplified and analysed by agarose gel electrophoresis. The full image of the gel was included in the supplementary information. (**C**) Sequencing results revealed that there was a deletion of 66 nucleotides in *PGAP2*-KO3.

To achieve this disruption, the *PGAP2* gene in HEK293 cells was knocked out using CRISPR/Cas9. PGAP2 is required for the addition of a saturated fatty acid to lyso-GPI-anchors during the GPI fatty acid remodelling reaction in the Golgi (Fig. 1A)^17^. Deficiency of this protein results in the transport of lyso-GPI-APs, which are sensitive to PLD. Consequently, significant parts of GPI-APs are secreted into the medium^17^. As expected, when the *PGAP2* gene was knocked out in HEK293 cells, surface expression of CD59, a ubiquitously expressed GPI-AP, was decreased significantly (Fig. 2A). The KO target region was amplified from the genome and sequenced, showing that there was the 66 bp deletion between target 1 and 2 sequences in the coding sequence (Fig. 2B,C). The deletion causes removal of 22 amino acids from the luminal region of the PGAP2, suggesting that this mutation inactivates protein function. Although the mutation is associated with hyperphosphatasia with mental retardation syndrome^24, 25^, *PGAP2*-KO cells did not show any growth and shape defects at the cellular level.

### Rescue of the *PGAP2* gene using *PB*-mediated insertion to the *PGAP2*-KO cells

**Figure 3:**
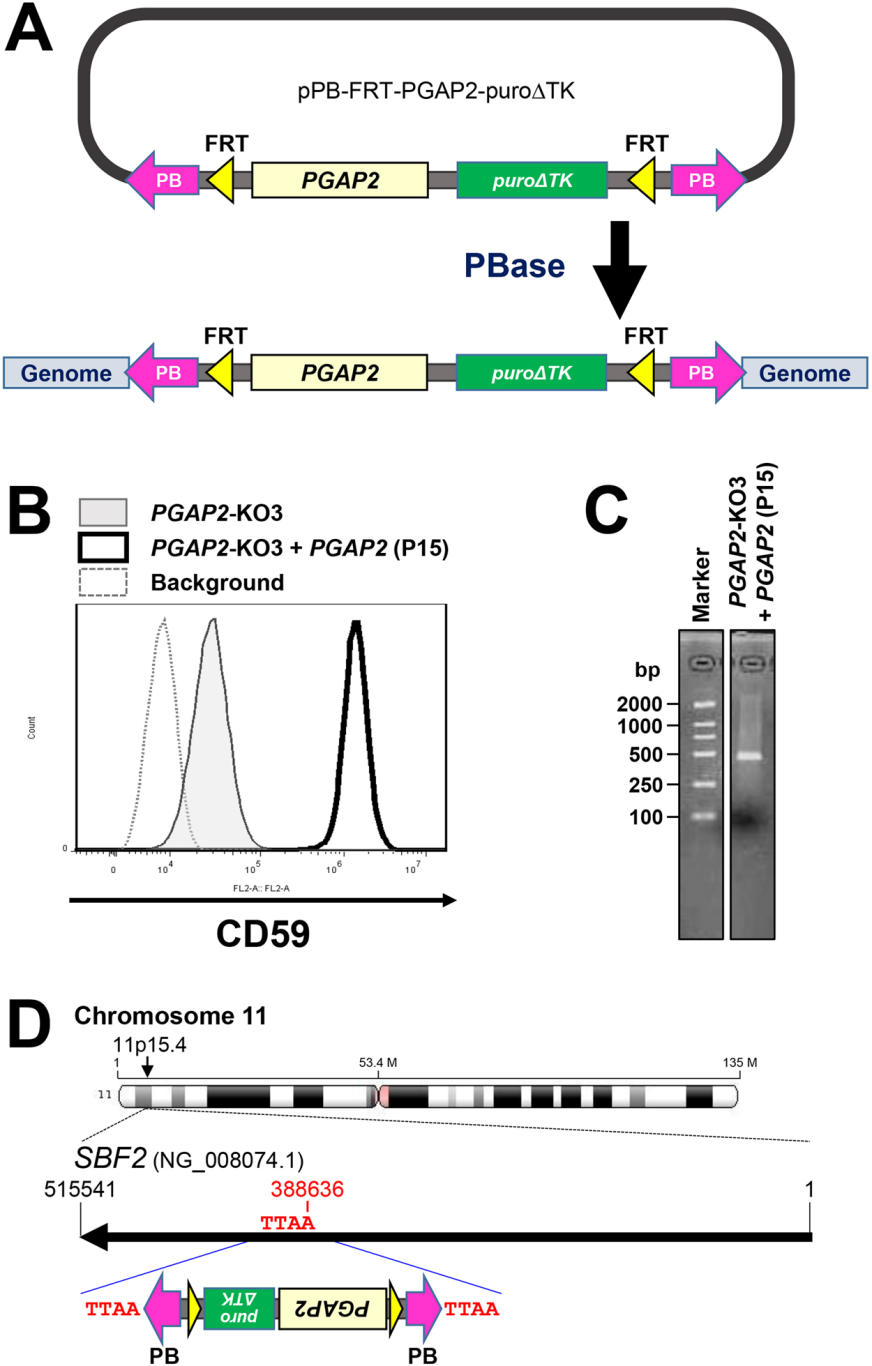
Restoration of CD59 surface expression after *PB*-mediated rescue of *PGAP2*. (**A**) Schematic view of *PGAP2* rescue using the *PB* transposon system. Plasmids containing *PGAP2* and *puroΔTK* flanked by *PB* and *FRT* at both ends were transfected into cells together with a plasmid expressing PBase. After puromycin selection, cells stably expressing *PGAP2* were obtained. (**B**) Restoration of surface expression in *PGAP2*-KO cells. *PGAP2*-KO3 cells were co-transfected with pCMV-hyPBase expressing *PB* transposase and pPB-FRT-PuroΔTK-PGAP2 to integrate the *PGAP2* gene into the genome. CD59 on the cell surface was detected by flow cytometry. (**C**,**D**) After genomic DNA was extracted from P15 cells, the *PB* insertion site and *PB* copy number were determined in the genome. In P15 cells, only one *PB* copy band was detected (**C**). The full image of the gel was included in the supplementary information. The *PB* element was inserted into the intron region of the *SBF2* gene region on chromosome 11 (**D**).

The *piggyBac* (*PB*) transposon system allows reversible transgenesis and precise re-excision from the genome^26, 27^. The *PGAP2* gene was rescued by a plasmid carrying the *PB* transposon. The plasmid pPB-FRT-PuroΔTK-PGAP2 construct includes the *PGAP2* gene and the *puroΔTK* selection cassette flanked by *PB* and *FRT* sequences (Fig. 3A). *PGAP2*-KO cells were co-transfected with pCMV-hyPBase expressing the *PB* transposase (PBase) and pPB-FRT-PuroΔTK-PGAP2 to integrate the *PGAP2* gene into the genome (Fig. 3A). After transfected cells were treated with puromycin, surviving cells were diluted and clonal cell lines were obtained. When the *PB* transposon is used for gene integration into the genome, several copies of the construct may be found in a cell^28^. However, a cell line that contains only one *PB* copy number in the genome was desired for further purposes. Among the clonal cells, we chose one cell line, named HEK293pB-PGAP2 clone P15 (P15), which rescued the expression of CD59 on the cell surface and had only one *PB* insertion site in the genome (Fig. 3B,C). The *PB* element containing the *PGAP2* gene was inserted at the TTAA sequence of the intron of the *SBF2* (SET Binding Factor 2) gene located on chromosome 11 (Fig. 3D).

**Figure 4:**
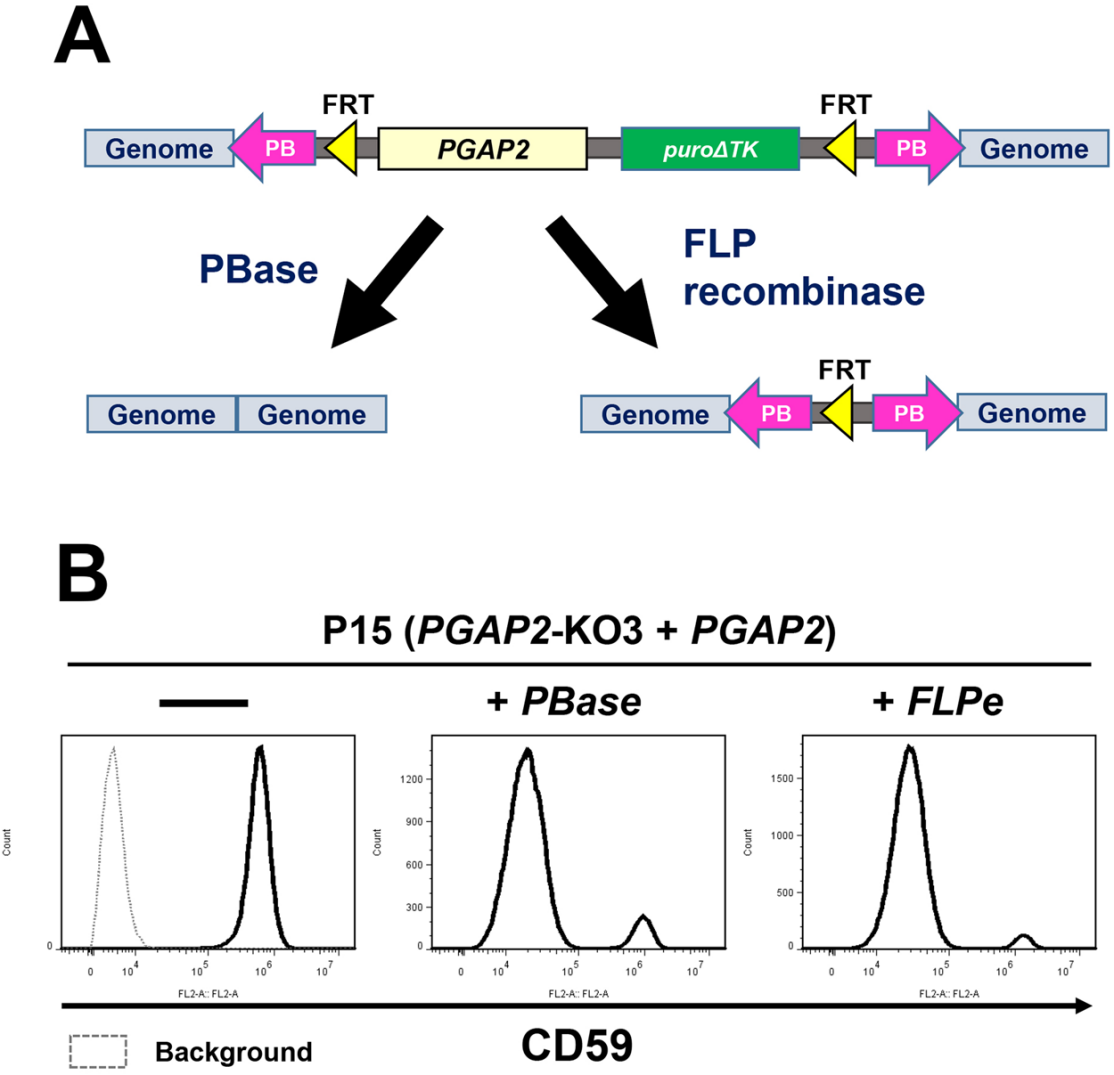
Re-excision of the *PB* transposon. (**A**) The *PB* element in the genome was re-excised by the expression of PBase or FLP recombinase. (**B**) Flow cytometric analysis of P15 cells after expression of PBase and FLP recombinase, followed by FIAU treatment. CD59 on the cell surface was detected.

Since the integrated *PGAP2* fragment is flanked by *FRT* sites and the *PB* transposon at both ends, the *PGAP2* gene can be re-excised by FLP recombinase or PBase (Fig. 4A). In addition, puroΔTK was used as the positive and negative selection marker for insertion. The cells excised with the *PB* element containing *PGAP2* and *puroΔTK* genes are resistant to treatment with the nucleoside analogue, 1-(2-deoxy-2-fluoro-1-D-arabinofuranosyl)-5-iodouracil (FIAU). After the plasmids expressing FLP recombinase or PBase were transfected into P15 cells, followed by FIAU treatment, the surface expression of CD59 on the surviving cells was analysed by flow cytometry. By expression of PBase or FLP recombinase, the P15 phenotype was reverted to that of *PGAP2*-KO cells, suggesting that the *PGAP2* gene was excised correctly (Fig. 4B). These data indicate that the P15 cell line can easily convert from GPI-positive cells to GPI-less cells by expression of PBase or FLP recombinase. By deletion of *PGAP2*, GPI-APs can be switched from membrane-bound forms to secreted forms.

### Expression of recombinant proteins using GPI anchors

**Figure 5:**
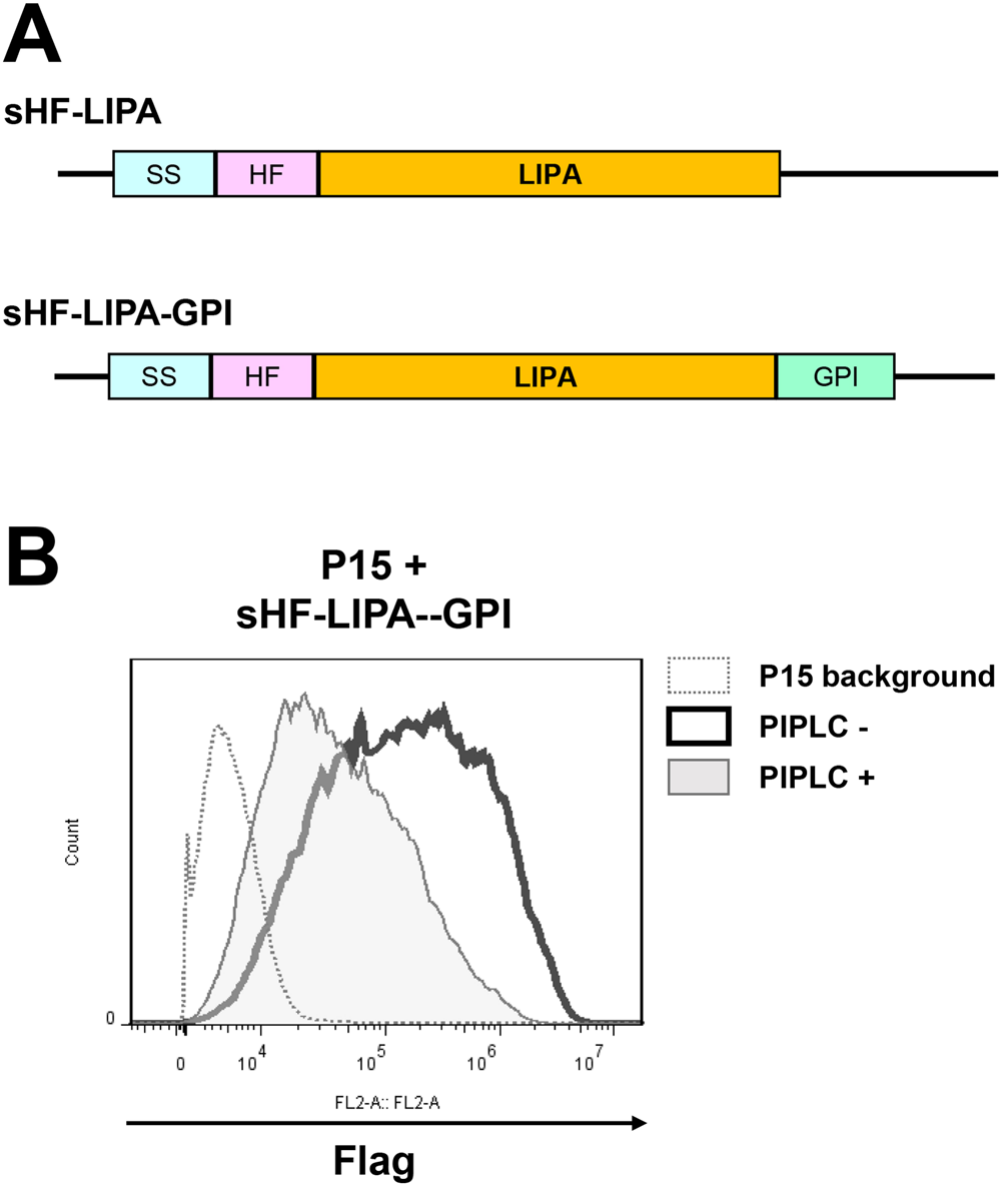
Expression of LIPA as a GPI-anchored form. (**A**) Schematic view of the plasmid constructs of the His6-Flag (HF)-tagged secretory LIPA (sHF-LIPA) and the GPI anchored LIPA (sHF-LIPA-GPI). The signal sequence (SS) and GPI attachment signal of human CD59 were used. (**B**) Flow cytometric analysis of sHF-LIPA-GPI on the cell surface of P15 cells. The cells were stained with an anti-Flag antibody and a PE-conjugated goat anti-mouse IgG. After PI-PLC treatment, surface expression of sHF-LIPA-GPI was found to decrease.

Recombinant proteins can be expressed as GPI-APs by simply conferring a GPI-attachment signal sequence to the carboxyl-terminus of the target protein. In the ER, the carboxyl-terminal GPI-attachment signal is exchanged with preformed GPI and this reaction is mediated by GPI transamidase^10^. As a model protein to express in this system, we chose lysosomal acid lipase (LIPA), which is an enzyme required for breakdown of lipids such as cholesterol esters and triacylglycerols in lysosomes^29^. Mutations in the LIPA gene cause lysosomal storage diseases such as Wolman disease and cholesteryl ester storage disease^30^. For treatment of LIPA deficiency, Kanuma (Sebelipase alfa), which is a recombinant LIPA produced by transgenic chicken egg, was approved for use in 2015 in the USA and EU^31^. We constructed vectors carrying His6-Flag-tagged LIPA (sHF-LIPA) or sHF-LIPA fused with a GPI-attachment signal (sHF-LIPA-GPI), which were stably expressed in cells (Fig. 5A). The sHF-LIPA-GPI was expressed on the cell surface and cleaved by treatment with PI-PLC (Fig. 5B), indicating that LIPA was expressed as a GPI-anchored form.

### Selection of cells highly expressing recombinant proteins

**Figure 6:**
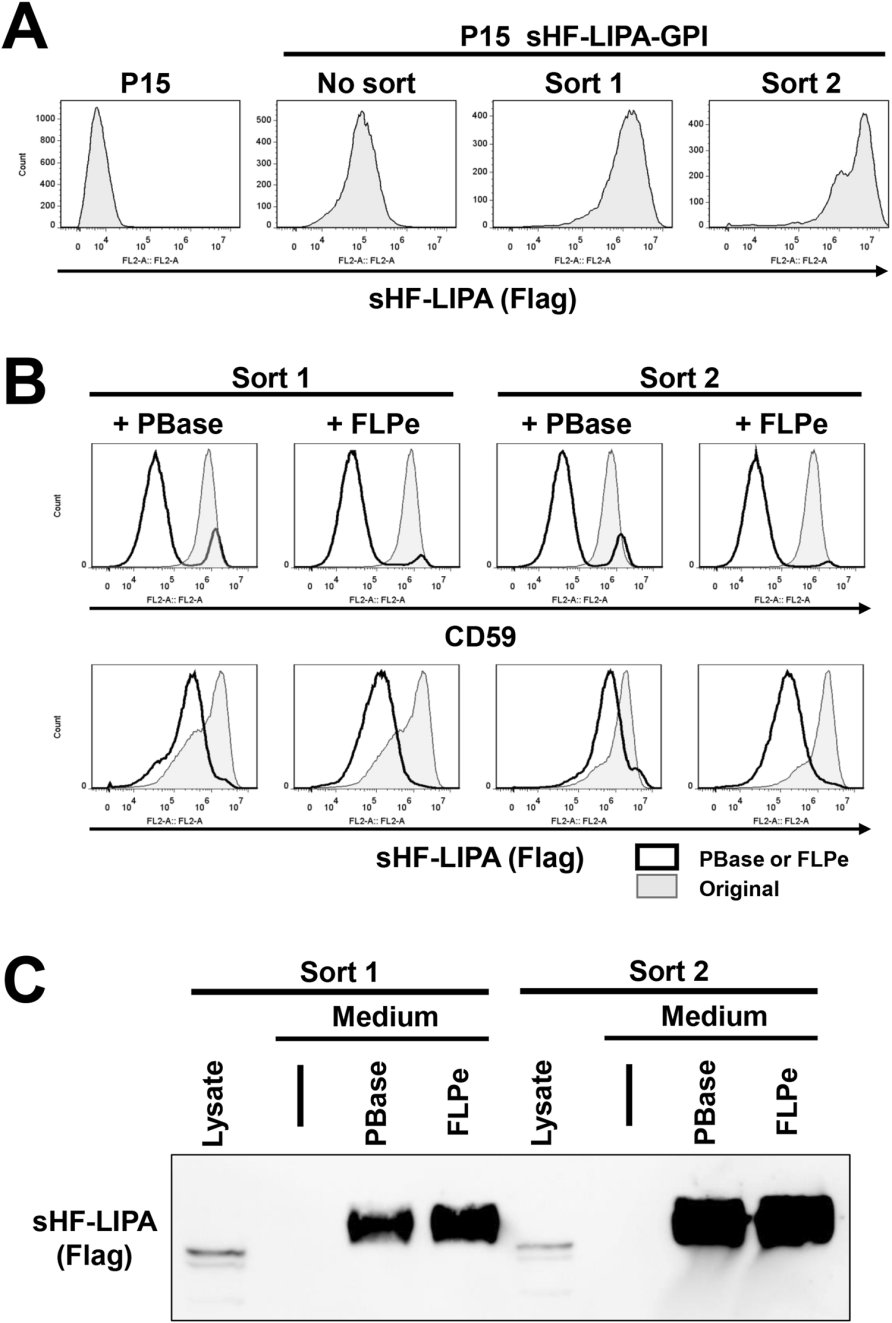
Enrichment of cells highly expressing LIPA. (**A**) Sorting of cells highly expressing sHF-LIPA-GPI. P15 cells stably expressing sHF-LIPA-GPI were sorted twice. Surface expression of sHF-LIPA-GPI was analysed by flow cytometry. Cells were stained with an anti-Flag antibody followed by a PE-conjugated goat anti-mouse IgG. (**B**) Sorted cells were transfected with the vector carrying PBase or FLPe recombinase. Expression of CD59 and sHF-LIPA-GPI on the cell surface was analysed. Original, P15 cells expressing sHF-LIPA-GPI after one round of sorting (Sort 1) and after two rounds of sorting (Sort 2). (**C**) sHF-LIPA in the cell lysate and culture medium was analysed. Culture medium from Sort 1 and Sort 2 cells with or without (−) treatment of PBase or FLPe recombinase were collected and precipitated. Proteins were detected by anti-Flag antibodies. The full image was included in the supplementary information.

The developed over-expression system enabled the sorting of cells expressing high amounts of LIPA. The plasmid for the expression of sHF-LIPA-GPI was transfected into P15 cells and the cells stably expressing sHF-LIPA-GPI were selected with antibiotics. The cells with high levels of expression of sHF-LIPA-GPI on the cell surface were then enriched using a cell sorter (Fig. 6A). After sorting twice, the expression of sHF-LIPA-GPI on the cell surface was increased by 28-fold (with the geometric mean fluorescent intensity) when compared with that of unsorted cells. The *PGAP2* gene was then removed from the sorted cells by expression of PBase or FLP recombinase. The surface expression of CD59 and sHF-LIPA-GPI decreased (Fig. 6B). Removal of the *PGAP2* gene from cells led to the secretion of sHF-LIPA into the medium (Fig. 6C), whereas P15 cells expressed sHF-LIPA-GPI on the cell surface but did not secrete this protein into the medium. Compared with cells that were sorted once, higher amounts of sHF-LIPA were secreted into the medium for cells that were sorted twice, suggesting that the system requires two sorting steps to ensure maximum selection and thus protein yields.

**Figure 7:**
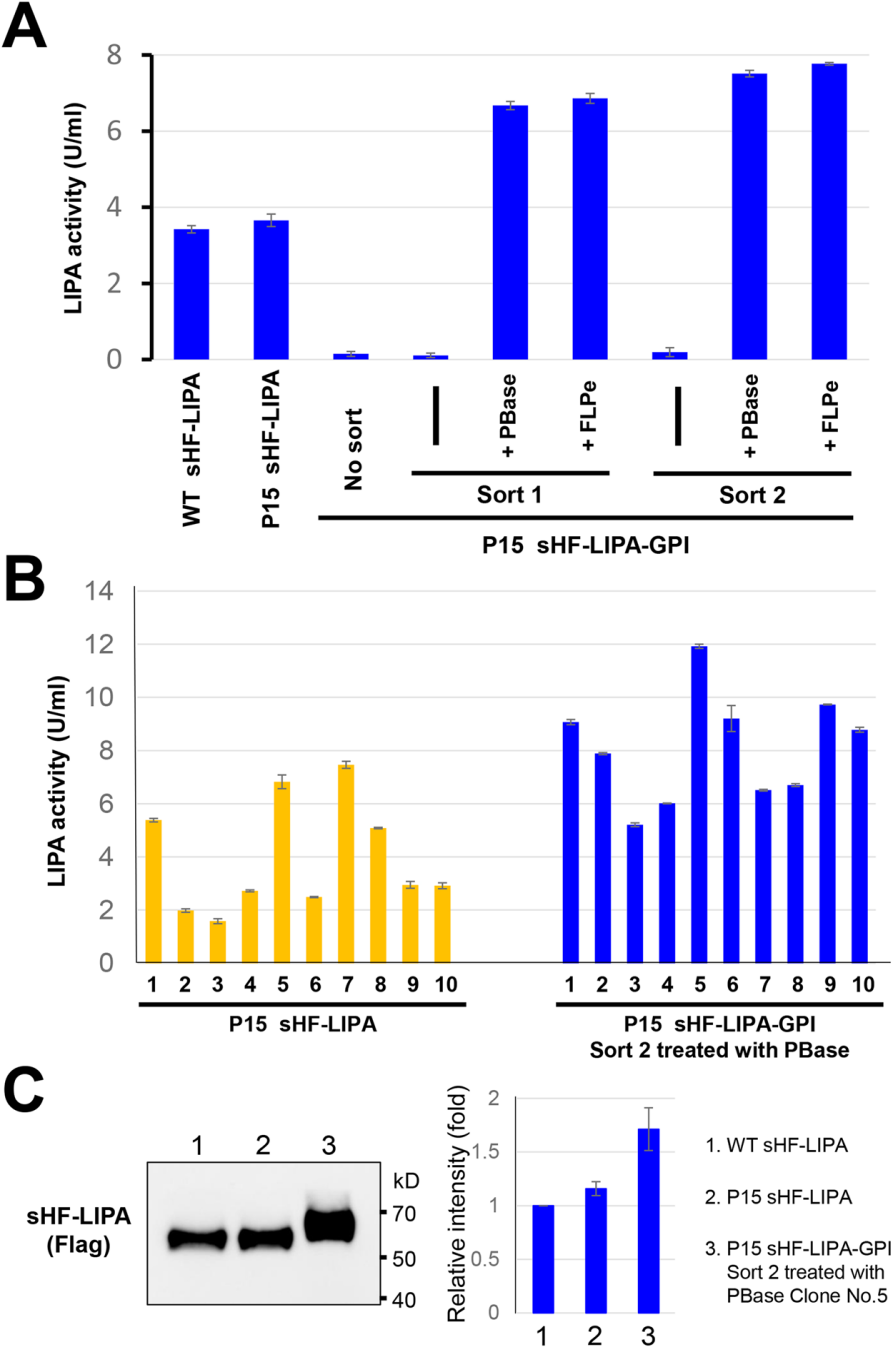
Secreted LIPA activities from P15 sorted cells. (**A**) LIPA activity using cell culture medium. The medium was collected after culturing 2.5 × 10^5^ cells for 48 h. LIPA activities in the medium were measured by conversion of 4-NPP to nitrophenol. The values shown are means ± SD of triplicate determinants. Results represent one of three comparable experiments. (**B**) LIPA activities in single clonal cells from P15 cells stably expressing soluble sHF-LIPA and PBase-treated P15 cells expressing sHF-LIPA-GPI after two rounds of sorting. The values shown are means ± SD of triplicate determinants. (**C**) Western blot of LIPA secreted from HEK293 (WT: 1) and P15 (2) cells stably expressing soluble sHF-LIPA and clone No. 5 of the PBase-treated P15 (3) cells expressing sHF-LIPA-GPI after two rounds of sorting (left). Culture media were collected and precipitated. Proteins were detected by anti-Flag antibodies. The full image was included in the supplementary information. The relative intensity of sHF-LIPA in the western blot was plotted. The intensity of the sHF-LIPA expressed from WT cells was set as 1. The values shown are mean ± error of two independent experiments.

LIPA activity was detected from the culture media of wild-type HEK293 cells and P15 cells expressing soluble sHF-LIPA (Fig. 7A). In contrast, the activity was not detected in the medium of P15 cells expressing sHF-LIPA-GPI without treatment. Once PGAP2 was removed by either PBase or FLP recombinase, however, a significant level of LIPA activity was detected (Fig. 7A). The activities from the first sorted cells and the second sorted cells were 1.8 and 2.0 times higher than that of P15 cells expressing soluble sHF-LIPA, respectively.

After the second sorting, we isolated clonal cell lines from P15 cells expressing sHF-LIPA-GPI by limiting dilution. We selected 10 clonal cell lines from P15 cells expressing sHF-LIPA and P15 cells expressing sHF-LIPA-GPI that were sorted twice followed by the removal of PGAP2. The sHF-LIPA was expressed in the medium at higher amounts in cells using the GPI-anchoring system when compared with that of the control P15 cells stably expressing soluble sHF-LIPA (Fig. 7B). We picked up one clonal cell line No. 5 that showed highest LIPA activity and detect the LIPA proteins. The protein levels secreted from clone No. 5 were increased 1.7 times compared to those from wild type cells expressing soluble LIPA (Fig. 7C). Therefore, the system can better and more easily select cells that are expressing recombinant proteins when compared with previous methods of secreting proteins into the medium.

### High expression of α-galactosidase A using GPI-based protein expression system

**Figure 8:**
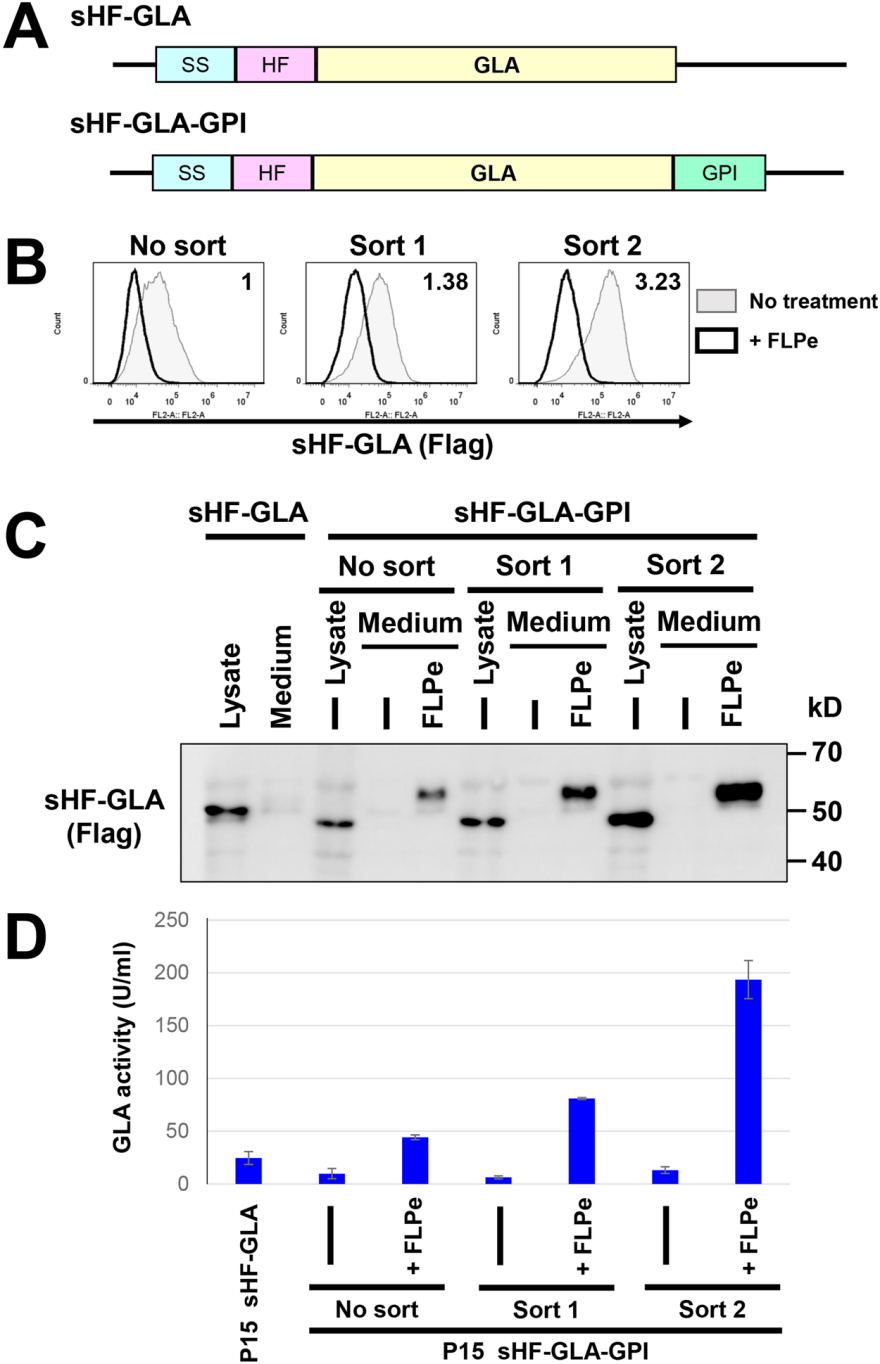
Selection of cells highly expressing GLA using the molecular switching system. (**A**) Schematic view of the plasmid constructs of the His6-Flag (HF)-tagged secretory GLA (sHF-GLA) and the GPI anchored GLA (sHF-GLA-GPI). The signal sequence (SS) and GPI attachment signal of human CD59 were used. (**B**) Sorting of cells highly expressing sHF-LIPA-GPI. P15 cells stably expressing sHF-GLA-GPI were sorted twice. Sorted cells were transfected with or without the vector carrying FLPe recombinase. Expression of sHF-GLA-GPI on the cell surface on the P15 cells expressing sHF-GLA-GPI before (No sort), after one round of sorting (Sort 1) or after two rounds of sorting (Sort 2) was analysed. Cells were stained with an anti-Flag antibody followed by a PE-conjugated goat anti-mouse IgG. (**C**) sHF-GLA in the cell lysate and culture medium was analysed. Culture medium from Sort 1 and Sort 2 cells with or without (−) treatment of FLPe recombinase were collected and precipitated. Proteins were detected by anti-Flag antibodies. The full image was included in the supplementary information. (**D**) GLA activity using cell culture medium. The medium was collected after culturing 2.5 × 10^5^ cells for 48 h. GLA activities in the medium were measured by conversion of MU-Gal to 4-methylumbelliferone. The values shown are means ± SD of triplicate determinants. Results represent one of two comparable experiments.

We finally analysed whether the system can be applied to express other recombinant proteins. As the protein to express, we chose the α-galactosidase A (GLA), which is another lysosomal enzyme. Recombinant GLA is used for the enzyme replacement therapy of the Fabry diseases^32^. Plasmids expressing His6-Flag-tagged GLA (sHF-GLA) and GPI-anchored sHF-GLA (sHF-GLA-GPI) were constructed and transfected into P15 cells (Fig. 8A). After selection by antibiotics, P15 cells expressing sHF-GLA-GPI were stained with anti-Flag antibodies and cells highly expressing GPI-anchored sHF-GLA on the cell surface were enriched twice by cell sorter. The surface level of sHF-GLA was increased 3.2 times (Fig. 8B). When *PGAP2* gene was removed from cells by the expression of FLPe recombinase, surface levels of sHF-GLA was decreased. Instead, the proteins started being released into the medium (Fig. 8C). The secreted GLA from cells sorted once and twice were higher than those from unsorted cells and cells expressing soluble sHF-GLA. The activity of GLA was increased in medium prepared from cells sorted twice (Fig. 8D). These results indicate that the system is useful to select cells highly expressing recombinant proteins of interest.

## Discussion

In this study, we developed a new system to select cells expressing high amounts of recombinant proteins. The advantage offered by GPI-anchoring of proteins was used to tether proteins to the cell surface^11, 19^. By enrichment of cells, in which higher amounts of GPI-anchored recombinant proteins are expressed on the cell surface, we could obtain cells expressing soluble target protein in high amounts. Here, we have established a HEK293 cell line, named P15, in which GPI-APs can be converted from membrane-bound forms to secretory forms. Initially, the *PGAP2* gene was knocked out in HEK293 cells to secrete GPI-APs into the medium. Second, the *PGAP2* gene was rescued in KO cells by a *PB* transposon-based plasmid. By introduction of *PB* transposase or FLP recombinase, the *PGAP2* gene can be easily removed from P15 cells, yielding *PGAP2*-KO cells once more. Using the P15 cells, recombinant proteins, LIPA and GLA, were expressed as the GPI-anchored form. After sorting cells that expressed high levels of GPI-anchored forms of proteins on the cell surface, LIPA-GPI and GLA-GPI were switched from the membrane-bound form to the secretory form by removal of the *PGAP2* gene from the cells. The system does not require treatment with any GPI-cleaving enzymes for release. Lyso-forms of GPI-APs are further cleaved by PLD-like activity^17, 18^. The activity arises from both endogenous expression in cells and the serum. With this method, we obtained cell lines that expressed recombinant LIPA and GLA at higher levels when compared with conventional methods.

When a GPI-attachment signal is added to the C-terminus of secretory proteins, the proteins are expressed as GPI-APs. Therefore, it has been possible to express a wide range of recombinant proteins on the cell surface through GPI-anchors^19, 33^. In particular, it would be useful to tether soluble proteins and type-I membrane proteins. GPI-anchored LIPA and GLA were functional, thereby showing that the GPI moiety did not hinder proper protein folding and function. Here, we used His6-Flag-tagged LIPA and GLA as model recombinant proteins to express, because such tags are useful for protein purification and detection. The system can also be used with non-tagged native proteins if an antibody against the target protein is available. In our system, target proteins expressed on the cell surface are stained with antibodies and the high expressing cells are enriched using flow cytometry-based cell sorters or magnetic separators. Therefore, it is a requirement that antibodies can be used with flow cytometry.

In this system, the GPI-glycan part is attached to the C-terminus of proteins, which has both advantages and disadvantages with respect to affecting the function and properties of the target protein. A clear disadvantage is that C-terminal labelling may lead to loss of protein function, and thus our system cannot be used for proteins that suffer loss of activity upon C-terminal modification^34^. In addition, because proteins are modified with the GPI-glycan, they are not identical to their native forms. Since soluble GPI-APs, which are cleaved by GPI-cleaving enzymes, exist in our body, cleaved GPI itself would not become the antigen. When the proteins are used for clinical purpose, however, attention should be paid to immunogenicity, because attachment of the GPI-glycan yields non-native target proteins. These negative points need to be addressed and improved in future studies.

Advantages of the expression system include protection from carboxypeptidases degradation because of C-terminal modification with a glycan moiety^35^. Second, there are several proteins that recognize GPI-glycans such as α-toxin and aerolysin^36, 37^, which can be used for enrichment and purification of target proteins^38^. Third, GPI-anchors have unique structures and are chemically and enzymatically processed^39, 40^. GPI moieties are also chemically modified *in vivo* ^41–43^. Therefore, the GPI-anchored recombinant proteins could be labelled or crosslinked with reagents such as fluorescent probes and nanoparticles. Further research would help to expand the applications of the system.

Plasmids expressing target proteins with monitoring proteins such as GFP are used for the selection of cells expressing recombinant proteins in high levels^44, 45^. In these methods, it is assumed that cells highly expressing monitoring proteins are also able to secrete large amounts of recombinant proteins. Since our method monitors the recombinant protein on the cell surface, it is a more direct approach to monitor protein expression levels. The dihydrofolate reductase (DHFR)-methotrexate (MTX) system is frequently used for increasing the expression of recombinant pharmaceutical proteins^46^. In this system, plasmids carrying a target gene of interest with *DHFR* are transfected into *dhfr*-deficient cells. By increasing the DHFR inhibitor MTX to select cells, the *DHFR* region together with target gene are amplified in the genome. Our system can be combined with the DHFR system to improve protein productivity. The advantage of our system is that cells expressing high levels of the target recombinant protein are enriched by cell sorting. Our findings show that the GPI-based protein expression system is more efficient when compared with other methods, as it is faster and highly productive. The system should be useful for large-scale production of recombinant proteins.

## Material and Methods

### Cells, antibodies and reagents

Human embryonic kidney 293 (HEK293) cells were cultured in Dulbecco’s Modified Eagle medium (DMEM) supplemented with 10% fetal calf serum. The appropriate antibiotic concentrations were used where necessary: puromycin (1 μg/ml), hygromycin (400 μg/ml), and streptomycin (100 U/ml)/penicillin (100 μg/ml). Mouse monoclonal anti-CD59 (clone 5H8), which was kindly provided from Taroh Kinoshita (Osaka University), and anti-M2 Flag antibodies (Sigma, St. Louis, MO, USA) were used as primary antibodies, whereas phycoerythrin (PE)-conjugated goat anti-mouse IgG (Biolegend, San Diego, CA, USA) was used as the secondary antibody. 1-(2-Deoxy-2-fluoro-β-D-arabinofuranosyl)-5-iodo-2,4 (1H,3H)-pyrimidinedione (FIAU) and 4-NPP were purchased from Sigma.

### Knockout of PGAP2 in HEK293 cells

The *PGAP2* gene was knocked out by the CRISPR/Cas9 system^47^. The pX330-EGFP plasmid vector was digested with *Bbs*I^48^. The PGAP2-KO target 5′-AGAAGCGAGGCGCCGAGTGC-3′ and 5′-GCGGGCGATAGCAGGAACAC-3′ were designed using the E-CRISP website (http://www.e-crisp.org/E-CRISP/)^49^ and were ligated into digested pX330-EGFP to generate pX330-EGFP-PGAP2-1 and pX330-EGFP-PGAP2-2, respectively. After transfection of knockout constructs into HEK293 cells, GFP positive cells were sorted using an S3e Cell Sorter (BioRad, Hercules, CA, USA). Sorted cells were further cultured for 10 d and limiting diluted to obtain the clonal cells. The knockout of *PGAP2* was confirmed by PCR.

### Plasmid construction

The plasmid pME-Puro-sHF-GPI containing a signal sequence, a His6 tag, a Flag tag and a GPI attachment signal of human CD59 were used to construct a plasmid for expression of GPI-anchored LIPA and GLA. LIPA and GLA fragments were obtained from human cDNA amplified using the primer sets 5′-CGATGACAAGCTCGAGGGGAAACTGACAGCTTTG-3′/5′-TCCCACCATTCTCGAGCTGATATTTCCTCATTAGATTAATAATT-3′ and 5′-CGATGACAAGCTCGAGCTGGACAATGGATTGGCAAGG-3′/5′-TCCCACCATTCTCGAGAAGTAAGTCTTTTAATGACATCTGCATTG-3′, respectively. The fragments were then ligated into the *Xho*I site of pME-Puro-sHF-GPI by In-fusion cloning (Takara, Shiga, Japan). The resulting constructs, pME-Puro-sHF-LIPA-GPI and pME-Puro-sHF-GLA-GPI, were confirmed by sequencing. The sHF-LIPA-GPI and sHF-GLA-GPI fragments were digested and ligated into the pME-Hyg plasmid to yield the pME-Hyg-sHF-LIPA-GPI and pME-Hyg-sHF-GLA-GPI plasmids, respectively.

For the plasmid expressing the soluble form of LIPA or GLA, a DNA fragment corresponding to the mature LIPA or GLA sequence after the ER insertional sequence was amplified and ligated into *Xho*I and *Not*I sites of pME-Hyg-sHF-LIPA-GPI to generate pME-Hyg-sHF-LIPA and pME-Hyg-sHF-GLA. For gene rescue mediated by the *PB* system, pPB-FRT-PGKp-PuroΔTK was constructed and contained a *PGK* promoter, a multiple cloning site, a bovine growth hormone (*GH*) poly-adenylation signal, *SV40* promoter and *puroΔTK* flanked by both *PB* terminal repeat sequences and flippase recognition target (*FRT*) at both ends. The Flag-tagged rat *PGAP2* gene was amplified and cloned into *Eco*RI and *Not*I of pPB-FRT-PGKp-PuroΔTK to give pPB-FRT-PuroΔTK-PGAP2. The pCMV-hyPBase was kindly provided from Kosuke Yusa (Sanger Institute) and pCAG-FLPe-IRES-puro was purchased from Addgene.

### Flow cytometry and cell sorting

Cells were harvested by a trypsin/EDTA solution for CD59 staining or by PBS containing 2 mM EDTA and 0.5% BSA for Flag staining. Cells (5 × 10^5^)/sample were stained with anti-CD59 or anti-Flag (10 μg/ml) as the first antibody, and PE-conjugated goat anti-mouse IgG was used as the second antibody. After staining, cells were analysed using Accuri C6 (BD, San Jose, CA, USA). Where necessary, cells were treated with phosphatidylinositol-specific phospholipase C (PI-PLC: Thermo Scientific, Carlsbad, CA, USA) for 1.5 h at 37 °C prior to antibody staining. For cell sorting, the cell sorting solution (PBS, 2 mM EDTA and 0.5% BSA) was used for resuspension of antibodies and washing instead of the FACS solution. Stained cells were sorted by an S3e Cell Sorter.

### PB copy number determination

To rescue *PGAP2*-KO cells, pPB-FRT-PuroΔTK-PGAP2 was co-transfected with pCMV-hyPBase. Two days after transfection, the cell medium was replaced by medium containing puromycin to select positive cells. After the limiting dilution, inserted copy number and insertion sites of the *PB*-*FRT*-*PuroΔTK*-*PGAP2* fragment were analysed for the obtained clones. The genomic DNAs from each clone were isolated using the Wizard genomic DNA purification kit (Promega, Madison, WI, USA). Genomic DNA was digested by *Hae*III, followed by ligation of adaptors. Splinkerette-PCR was performed to determine PB insertion sites^50, 51^. Flanking sequences were amplified using primer sets Spl-P1, 5′-CGAATCGTAACCGTTCGTACGAGAA-3′ and 3PB-1st, 5′-TATACAGACCGATAAAACACATGCGT-3′ for the 1st PCR, and Spl-P2 5′-TCGTACGAGAATCGCTGTCCTCTCC-3′ and 3PB-2nd, 5′-CGCATGATTATCTTTAACGTACGTCACAA-3′ for the second nested PCR. To determine PB copy number, amplified DNA was applied on 2% agarose gel and checked by electrophoresis. The insertion sites were determined by sequencing. The cell line, named P15, with a single PB copy in the genome was chosen for further experiments.

### Recombinant mammalian protein expression

To make stable cell lines expressing soluble forms sHF-LIPA or sHF-GLA and membrane-bound forms sHF-LIPA-GPI or sHF-GLA-GPI, linearized DNA plasmids were transfected into cells. Two days after transfection, cells were selected by culturing in medium containing hygromycin (400 μg/ml). To remove PB transposons from cells expressing GPI-fused proteins, sorted cells in a 6-well plate were transfected with 4 μg DNA of pCMV-hyPBase or pCAG-FLPe-IRES-puro for PBase or Flippase, respectively. After 2 d, medium containing 1 μM FIAU was applied, and the medium was changed every day for 7 d.

### Western blotting of sHF-LIPA and sHF-GLA prepared from medium and cell lysates

Cells (5 × 10^5^) were plated and cultured in 2 ml of medium. After 48 h, 1.4 ml medium was collected. The medium was centrifuged at 10,000 × *g* for 5 min and 1000 μl of the supernatant was collected in a new tube. Then, 20 μl of prewashed anti-Flag beads (MBL, Aichi, Japan) for DDDDK-tagged proteins were added. The tube was rotated at 4 °C for 2 h, and then centrifuged at 10,000 × *g* for 1 min. The supernatant was removed and the beads with tagged proteins were washed 3 times with cold PBS. The proteins were eluted with the Flag peptide. For the cell lysates, after removing the medium, 5 × 10^5^ cells were harvested with the cell sorting solution and washed using cold PBS at 4 °C. One hundred microliters of lysis buffer (50 mM Tris-HCl (pH 7.5), 150 mM NaCl, 1% Triton-X100, 1 mM EDTA, protein inhibitor, 1 mM PMSF) was added to the cell pellets and the mixture was incubated on ice for 30 min. After incubation, the tube was centrifuged at 10,000 × *g* for 15 min at 4 °C. The supernatant was collected and mixed with sample buffer and boiled at 95 °C for 5 min. The solution was kept at −20 °C until western blotting. The recombinant LIPA and GLA were detected using the anti-Flag antibody and horseradish peroxidase (HRP)-conjugated anti-mouse IgG as the primary antibody and secondary antibody, respectively. Proteins were visualized using ImageQuant^TM^ LAS 4000 (GE Healthcare, Little Chalfont, UK).

### LIPA activity

The medium was collected after culturing 2.5 × 10^5^ cells in a 12-well plate for 48 h in 1 ml medium without any antibiotics. After centrifugation at 10,000 × *g*, 0.8 ml of medium was collected and then stored at −20 °C until use. LIPA activity was measured by an optimized assay for lipase activity using cell medium^52, 53^. Briefly, 100 ml assay buffer (100 mM citrate buffer (pH 5.0), 0.5% Triton X-100, 1 mM CaCl_2_) and 20 μM 4-nitrophenyl-palmitate (4-NPP) dissolved in isopropanol/acetonitrile (4/1 v/v) were prepared before the assay. The assay mixture was obtained by adding 4-NPP to the assay buffer to give a final concentration of 4-NPP of 1 μM. The mixture was then placed in a 60 °C water bath for 5 min, mixing continuously until the solution was transparent. An appropriate amount of medium was centrifuged at 10,000 × *g* at 4 °C for 5 min, and the supernatant was collected in a new tube. Ten microliters of cell medium was added to 60 μl assay mixture in transparent 96-well plates, which was immediately covered by a dark paraffin paper before placing it in the incubator at 30 °C for 45 min. Subsequently, 120 μl of 1 M Tris–HCl (pH 8.0) was added to the assay mixture to terminate the reaction. The absorbance was measured at 405 nm, using an Enspire 2300 Multilabel Reader (Perkin Elmer, Waltham, MA. USA). One unit of lipase activity was defined as the amount of 4-nitrophenol nmol per min released from 4-NPP at 30 °C.

### GLA activity

The medium for GLA activity was prepared the same as described above for LIPA activity. For measuring GLA activity, an optimized method using 4-methyumbelliferyl-α-D-galactopyranoside (MU-Gal, Santa Cruz Biotechnology, Santa Cruz, CA, USA) as a substrate was described previously^54–56^. Briefly, 100 ml of assay buffer (60 mM citrate-phosphate buffer (pH 4.6), 1 mM MgCl_2_) and 20 mM MU-Gal dissolved distilled water were prepared before the assay. The assay mixture was obtained by adding MU-Gal to the assay buffer to make 2 mM MU-Gal solution. Ten μl of cell medium were added to 100 μl assay mixture in a dark 96-well plate, which was immediately covered by a dark paraffin paper before placing it in the incubator at 37 °C for 60 min. Afterwards, 100 μl of 0.4 M glycine-NaOH buffer (pH 10.6) were added to the assay mixture to terminate the reaction. The fluorescence was recorded using Enspire 2300 Multilabel Reader (Perkin Elmer, Waltham, MA, USA) at 350 nm and 465 nm for excitation and emission wavelengths, respectively. The 4-methylumbelliferone (Sigma) was used to create the standard curve. One unit of galactosidase activity was defined as the amount of 4-methylumbelliferone pmol per min released from MU-Gal at 37 °C.

## Electronic supplementary material

Supplementary Information
