# Supplementary material for: Molecular switching system using glycosylphosphatidylinositol to select cells highly expressing recombinant proteins
Source: Sci Rep. 2017 Jun 22;7:4033. doi: 10.1038/s41598-017-04330-3 (PMC5481379; doi:10.1038/s41598-017-04330-3)

Supplementary Information

Molecular switching system using glycosylphosphatidylinositol to select cells highly expressing recombinant proteins

Emmanuel Matabaro[1], Zeng'an He[1], Yi-Shi Liu[1], Hui-Jie Zhang[1], Xiao-Dong Gao[1], & Morihisa Fujita[1,*]

[1]Key Laboratory of Carbohydrate Chemistry and Biotechnology, Ministry of Education, School of Biotechnology, Jiangnan University, 1800 Lihu Avenue, Wuxi, Jiangsu 214122, China

Figure 2B

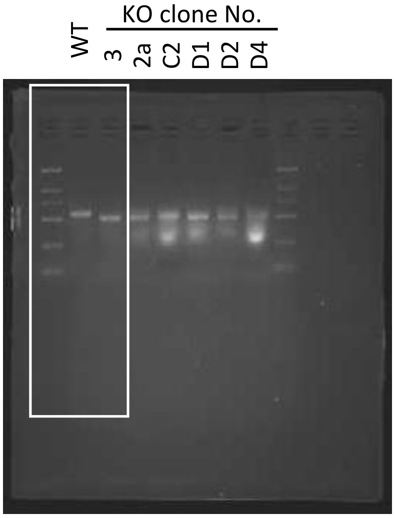

Figure 3C

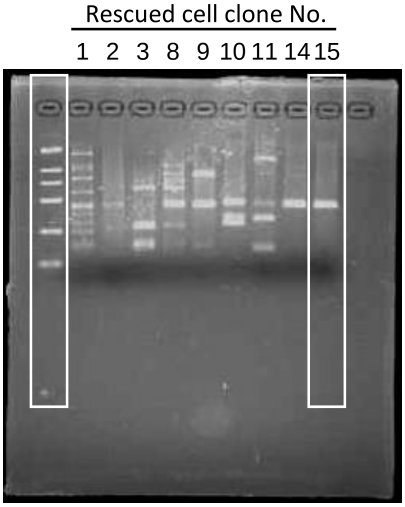

Figure 6C

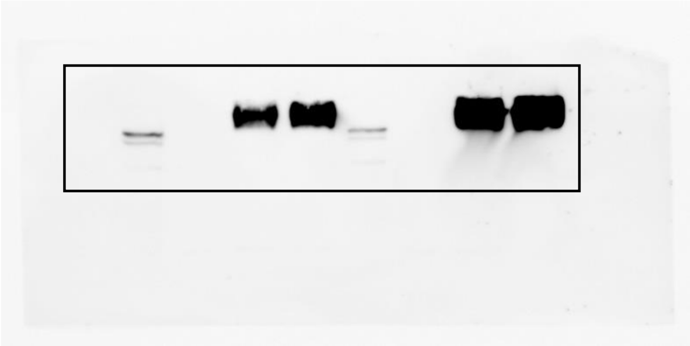

Figure 7C

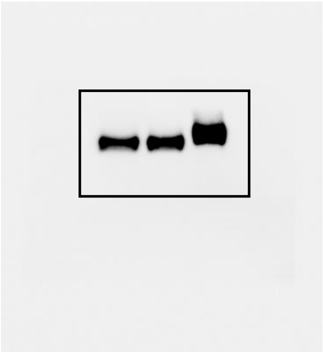

Figure 8C

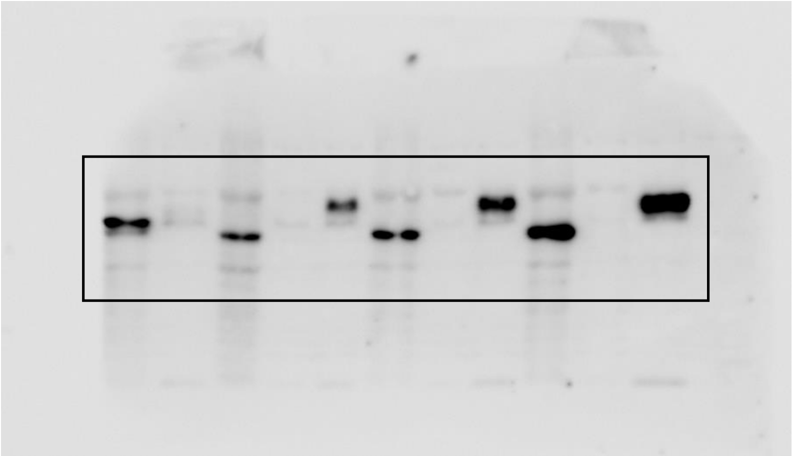

Supplement: Supplementary file 1 — Supplementary Information [file 41598_2017_4330_MOESM1_ESM.pdf]
